# Multiplex PCR Fluorescence Method for Detection of Genetically Modified Maize Strains

**DOI:** 10.3390/cimb48070677

**Published:** 2026-06-30

**Authors:** Wenxiu Yin, Wenxin Zhang, Quan Zhang, Zhengping Ying, Shan Wu, Huizhen Yu, Mingzhe Zhang

**Affiliations:** 1 Species Identification Laboratory, Zhejiang Academy of Science and Technology for Inspection and Quarantine, Hangzhou 310008, China; 2 Faculty of Science, The University of Melbourne, Melbourne 3000, Australia

**Keywords:** high-throughput screening, capillary electrophoresis, transgenic maize (*Zea mays* L.), event-specific identification, food safety monitoring

## Abstract

The rapid proliferation of genetically modified (GM) crops and the uncontrolled distribution of GM-based food and feed have become a growing global concern, posing new challenges for regulatory oversight and traceability. The traditional PCR detection method cannot simultaneously meet the needs of high-throughput, high-specificity and high-sensitivity detection of transgenic organisms. In this study, a multiplex fluorescence PCR-capillary electrophoresis platform was developed by labeling primers of endogenous and exogenous genes with different fluorescent groups. The system enabled the simultaneous detection of 27 GM-related genes and events in a single analytical workflow. The results demonstrated accurate identification of all seven GM maize events, with correct detection achieved for each individual strain. In addition, the method enabled precise discrimination of a mixed sample containing five GM maize varieties. The assay also achieved a detection sensitivity of 0.1% in gradient mixtures with different GM contents. Our platform integrates a larger number of targets into a single PCR reaction, thereby simplifying the detection workflow while maintaining high analytical performance. Furthermore, the combination of multicolor fluorescence labeling and capillary electrophoresis provides high-resolution fragment discrimination and robust multiplex detection capability. This platform provides a novel and effective tool for rapid detection in food safety of transgenic crops and related areas, and can be applied in import/export inspection, quarantine, and biosafety surveillance.

## 1. Introduction

Genetically modified (GM) crops exhibit higher yield and nutritional quality compared to conventional crops, while also enabling a certain reduction in pesticide use [1,2]. Furthermore, they possess superior tolerance to both biotic and abiotic stresses relative to conventional crops [3]. The production of genetically modified crops has experienced an exponential increase, rising nearly a hundredfold from 1996 to 2020 [4,5]. To ensure the safe and sustainable development of GM crops and to meet consumer demands for transparency and choice, countries worldwide have established comprehensive genetically modified organism (GMO) safety monitoring and evaluation systems [6].

The detection of genetically modified products is primarily divided into nucleic acid-based and protein-based techniques [7,8]. Protein-based detection methods have led to the development of specific monoclonal and polyclonal antibodies [9,10]. However, these methods cannot detect non-coding sequences, such as promoters or terminators [11]. Since different GM species possess distinct genetic sequences, DNA serves as the gold standard for GM crop detection [12]. In recent years, GMO detection has progressively shifted toward more sensitive, precise, and high-throughput analytical methods, such as digital PCR, next-generation sequencing, and CRISPR-based assays, to meet the increasing complexity of regulatory monitoring [8,13,14,15,16]. Despite these advances, PCR-based methods remain the most widely adopted tools for routine GMO screening because of their high sensitivity, reliability, low cost, and compatibility with established regulatory frameworks [17,18].

Among all GM crops, GM maize accounts for 32.5% of the total GM crop area, ranking second only to GM soybean and representing one of the most important GM crops [19]. Currently, many countries have approved the import and export of GM crops. Within the strict regulatory framework governing the commercial planting of GM maize, further refining line-specific detection methods to achieve rapid and accurate identification is imperative for strengthening biosafety management and safeguarding the public’s right to know regarding food safety.

However, with the rapid increase in detection throughput, conventional PCR can no longer meet the requirements of high-throughput testing, making it urgent to establish more efficient and accurate detection methods. For GM organism detection, the simultaneous detection of multiple gene elements is more advantageous and efficient than single-target assays. According to previous studies, the Cauliflower Mosaic Virus 35S (*CaMV35S*) promoter and the nopaline synthase terminator (*NOS*) terminator are the two most important GM detection elements used around the world [20]. They were used in 65.7% and 53.49% of all the commercialized GM organisms, respectively, and either or both were used in 81.4% of all GM events [19]. Thus, the development of a multiplex platform for GM detection will greatly facilitate the improvement of GM regulation.

Multiplex PCR, which allows multiple nucleic acid fragments to be amplified in a single reaction system using more than two pairs of primers, effectively addresses the challenges posed by the large number and diversity of GM events [15,21,22,23]. In this study, we established a high-throughput multiplex real-time PCR fluorescence detection method for transgenic maize events. Multiple pairs of specific detection primers targeting endogenous, exogenous, and event-specific genes were fluorescently labeled, and detection was performed by combining multiplex PCR amplification with capillary electrophoresis fluorescence. This approach achieves higher specificity, accuracy, sensitivity, and throughput than traditional methods.

While maintaining accurate identification, the multiplex design increased the detection throughput to 27 targets per reaction, far exceeding that of conventional real-time PCR. This greatly enhances detection efficiency without compromising reliability. Verified by repeated experiments, the detection sensitivity reached 0.1%, with stable and dependable results, providing a solid technical basis for the accurate qualitative and quantitative analysis of low-level GM components. In addition, the separation mode of multi-color fluorescent labeling coupled with capillary electrophoresis offers a superior capability to distinguish primer dimers and non-specific amplification products, thereby reducing the risk of false positives and false negatives. The method is particularly suitable for the analysis of complex matrices such as food and feed samples, and it meets the demands of entry-exit inspection and quarantine as well as food safety monitoring. Its high-throughput nature is especially advantageous for the rapid screening of large numbers of samples at ports and quality inspection agencies, effectively improving regulatory efficiency and ensuring smooth trade.

## 2. Materials and Methods

### 2.1. Sampling and DNA Extraction

For the four most popular GM crops—maize (*Zea mays* L.), soybean (*Glycine max* L.), rice (*Oryza sativa* L.), and rapeseed (*Brassica napus* L.)—we prepared different strains. Non-GM strains were collected from the market. The GM strains included seven maize strains (GA21, Bt176, Mon88017, Bt11, MON863, TC1507, NK603), three soybean strains (GTS-40-3-2, Mon89788, CV127), and three rapeseed strains (Ms8, Rf3, Rt73) from European Standards, and one rice strain (TT51) from the Chinese Academy of Inspection and Quarantine.

Then we employed the Dneasy Plant Mini Kit (Qiagen, Hilden, Germany) to extract DNA from seeds of both non-GM and GM strains. And the concentration is measured by Nano Drop 3300 nucleic acid analyzer (Thermo Fisher Scientific, Wilmington, DE, USA).

### 2.2. Primer Designation

When designing primers, the amplification specificity is verified using the BLAST function in GenBank v 265.0. Since primers from different groups may interfere with each other to some extent in a multiplex PCR amplification system, primer design must consider the interference and balance among primer pairs, and appropriate adjustments should be made. One of the two primers in each group is labeled at the 5′ end with a fluorescent marker selected from 6-FAM, HEX, and TAMRA, and each group is assigned a unique marker color (Table 1, full information in Supplementary Table S1).

### 2.3. Multiplex PCR Amplification

The DNA extracted from genetically modified samples was used as the template, and several pairs of primers were added for multiplex PCR amplification. The amount of every multiplex PCR primer was increased and the amount of TaqDNA polymerase was better adjusted in order to build an optimal reaction system. The PCR reaction system was as follows: Reaction Mix 10 μL; a warm start C-Taq 1 μL; Primers 5 μL; Template DNA 1 μL (mass concentration: ~30 ng/μL); add ddH_2_O to 25 μL (Table 2, full information in Supplementary Table S2). Amplification procedure was as follows: pre-denaturation at 95 °C for 2 min; pre-denaturation at 94 °C for 30 s; annealing at 59 °C for 50 s; extension at 72 °C for 50 s; 30 cycles; extension at 72 °C for 10 min.

### 2.4. Fluorescence Detection

PCR products (1 μL) were mixed with 12.5 μL of a sample mixture containing deionized formamide and AGCU Marker SIZ-500 (AGCU ScienTech Inc., Wuxi, China) [24] (12 μL and 0.5 μL per sample, respectively). After denaturation at 95 °C for 3 min and immediate cooling on ice, fragments were separated on an Applied Biosystems 310 Genetic Analyzer (Applied Biosystems, Foster City, CA, USA) using a 47-cm × 50-μm fused-silica capillary and the GS STR POP-4 (1 mL) G5 run module. Electrokinetic injection was performed at 15 kV, and electrophoresis was carried out at 60 °C for 25 min. Fluorescent fragments labeled with FAM, HEX, and TAMRA were detected using the DS-30 dye set and analyzed with GeneMapper software v4.1.

## 3. Results

### 3.1. High-Throughput Detection

Genetically modified testing sites are generally classified into three categories: endogenous genes, exogenous genes, and strain-specific genes. In this study, we focused on 27 target genes, including four endogenous reference genes from maize, soybean, rapeseed, and rice, eight exogenous genes, and fifteen GM strain-specific genes.

**Figure 1:**
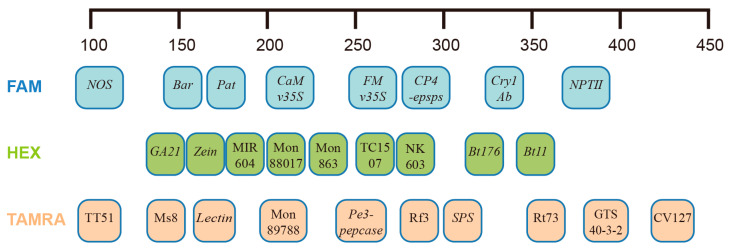
Gene sites for GMO detection.

The FAM channel was used to detect eight exogenous genes, including *NOS*, *Bar*, *Pat*, *CaMV35S*, *FMv35S*, *CP4*-*epsps*, *Cry1Ab* and *NPTII*. The HEX channel was used to detect the maize endogenous gene *Zein* and eight genetically modified maize events, including GA21, MIR604, Mon88017, MON863, TC1507, NK603, BT176 and Bt11). The TAMRA channel was used to detect the soybean endogenous gene *Lectin*, the rape endogenous gene *Pe3-pepcase*, and the rice endogenous gene *Sps*, the GM rice event TT51, three GM rapeseed events (Ms8, Rf3, and Rt73), and three GM soybean events (GTS-40-3-2, MON89788, and CV127) (Figure 1).

### 3.2. Specificity Analysis

**Figure 2:**
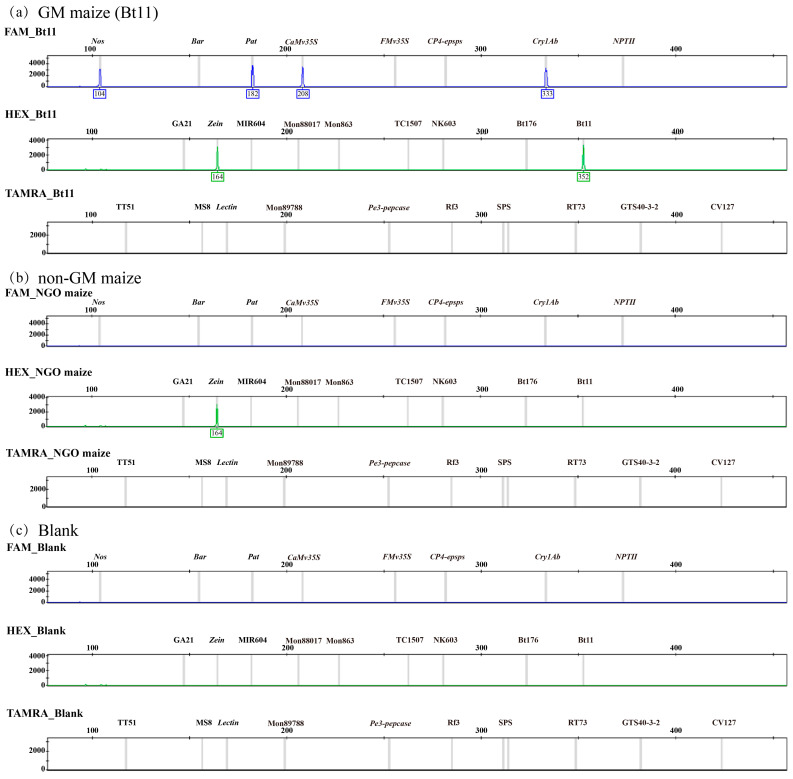
Polymorphic fingerprints of genetically modified (GM) Bt11. (**a**) Bt11 strain; (**b**) Non-genetic modified maize; (**c**) Blank.

We performed specificity analysis of the multiplex PCR fluorescence detection method using seven GM maize strains. As shown for the Bt11 strain (Figure 2a), clear amplification peaks were observed at the loci corresponding to the terminator gene *NOS*, the herbicide-resistance gene *Pat*, the promoter gene *CaMV35S*, the insect-resistance gene *Cry1Ab*, the endogenous maize reference gene *Zein*, and the strain-specific loci Bt11. No amplification signals were detected at other target loci. In addition, successful detection was also achieved for the other six strains (Supplementary Figures S1 and S2). Across all seven events, all expected targets were correctly identified (7/7, 100%). These results confirmed the successful identification of the Bt11 genetically modified maize strain and demonstrated the high specificity of the multiplex PCR fluorescence detection method for GMO detection.

### 3.3. Detection and Analysis of Mixed Strains

**Figure 3:**
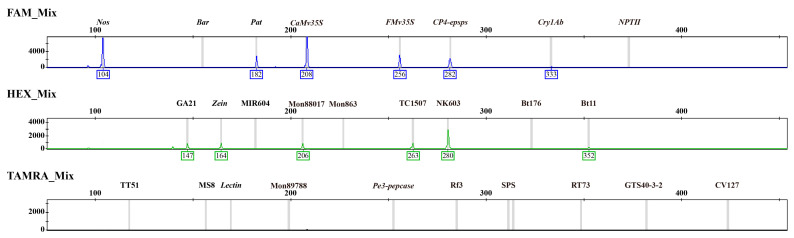
Polymorphic fingerprints of mixed genetically modified maize events.

The multiplex PCR fluorescence detection method was further applied to detect mixed genetically modified maize strains, including GA21, MON88017, TC1507, NK603, and Bt11. The FAM and HEX channels generated distinct amplification peaks at the target loci of the terminator gene *NOS*, the herbicide-resistance gene *Pat*, the promoter genes *CaMV35S* and *FMV35S*, the herbicide-tolerance gene *CP4-epsps*, the strain-specific loci GA21, MON88017, TC1507, NK603, and Bt11, as well as the endogenous maize reference gene *Zein* (Figure 3). No amplification signals were detected at other target loci. These results confirmed the successful simultaneous detection of the mixed genetically modified maize strains and demonstrated the reliability and specificity of the multiplex PCR fluorescence detection method for the identification of multiple GMO events.

### 3.4. Sensitivity Analysis

**Figure 4:**
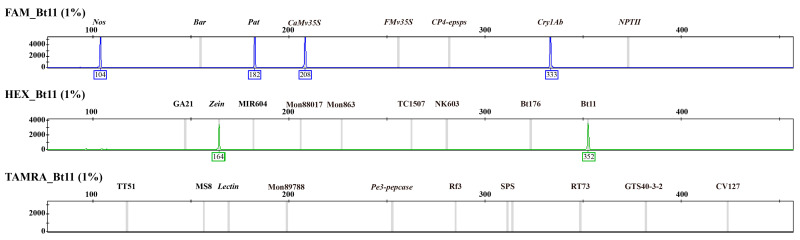
Polymorphism detection of mixed samples with transgenic (1%) and non-transgenic component.

**Figure 5:**
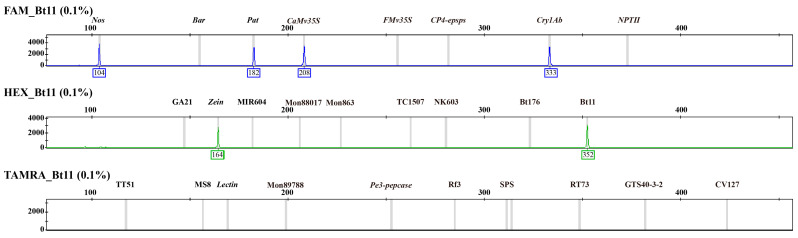
Polymorphism detection of mixed samples with transgenic (0.1%) and non-transgenic component.

**Figure 6:**
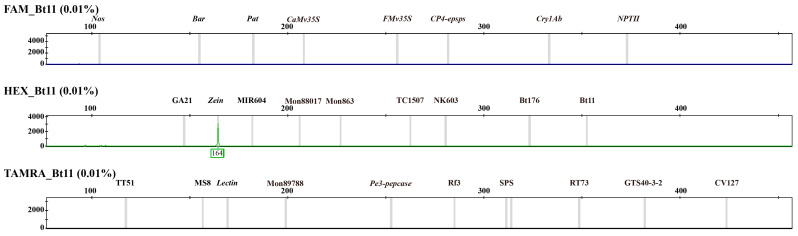
Polymorphism detection of mixed samples with transgenic (0.01%) and non-transgenic component.

To evaluate the sensitivity of the multiplex PCR fluorescence detection method, mixtures of non-genetically modified maize and genetically modified maize were prepared at GM proportion of 1%, 0.1%, and 0.01%. The fluorescence signals of the PCR products were subsequently analyzed. The amplification signal of the endogenous reference gene *Zein* remained stable across all tested concentrations. For the exogenous genes *CaMV35S*, *Pat*, *NOS*, and *Cry1Ab*, as well as the strain-specific loci Bt11, clear positive amplification signals were detected in samples containing 1% and 0.1% GM maize samples (Figure 4 and Figure 5). In contrast, no positive amplification signals were observed in samples containing 0.01% GM maize (Figure 6). Repeated experiments produced consistent results for the 0.1% GM samples, demonstrating the reproducibility of this method. Therefore, the detection limit of the multiplex PCR fluorescence detection method was determined to be 0.1% GM content.

## 4. Discussion

To meet the requirements of GMO legislation, the development of analytical tools for the detection of genetically modified organisms has become increasingly important. A range of complementary detection strategies has been established for GMO analysis, including PCR-based assays, immunochemical methods, genosensors, and next-generation sequencing (NGS)-based approaches, thereby expanding the analytical toolbox available for regulatory monitoring and surveillance [8,14].

Among these, PCR-based methods remain the most widely adopted in routine GMO screening due to their high sensitivity, reliability, low cost, and compatibility with established regulatory frameworks. Within PCR-based strategies, multiplex PCR coupled with capillary electrophoresis has become a key platform for high-throughput GMO detection [23,25,26,27]. Compared with qPCR and ddPCR (digital droplet PCR), which are generally applied for low- to moderate-plex target detection, multiplex PCR–capillary electrophoresis allows simultaneous detection of multiple GM targets within a single analytical workflow, making it particularly suitable for high-throughput screening applications. A previous study has organized multiple targets of GM maize into several independent multiplex reactions (e.g., 4–10-plex per panel), followed by capillary electrophoresis for fragment separation and detection [22]. Although such multi-panel designs improve screening capacity compared with singleplex PCR, they inevitably increase assay fragmentation and experimental complexity due to the requirement of multiple independent amplification reactions per sample.

In this study, we developed an expanded multiplex fluorescent PCR–capillary electrophoresis system capable of simultaneously detecting 27 GM-related targets, including endogenous reference genes, regulatory elements, and event-/strain-specific markers. Unlike multi-panel approaches, all targets were integrated into a single PCR reaction per sample, thereby reducing assay fragmentation and simplifying the overall workflow. This design reduces hands-on operation and improves laboratory efficiency while maintaining high analytical performance, with a detection sensitivity of 0.1%. Compared with previously reported systems, the present method offers a more integrated and streamlined platform for routine high-throughput GMO screening.

Event-specific detection is considered the most reliable strategy for GMO identification because it targets the unique junction sequence between the inserted transgenic cassette and the host plant genome [28,29]. Since each transformation event possesses a specific insertion site, event-specific assays provide high specificity and accuracy and have therefore been widely adopted in international GMO testing standards and regulatory laboratories.

In this study, the incorporation of event-/strain-specific markers, together with endogenous reference genes and common regulatory elements, enabled comprehensive GMO characterization within a single assay. The successful discrimination of different GM maize events and the accurate identification of mixed samples containing five GM maize varieties further demonstrated the high resolution and strong anti-interference capability of the platform. In addition, the system maintained a detection sensitivity of 0.1%, indicating its suitability for detecting low-level GM materials that may be present in commercial products.

Several limitations of the present platform should be acknowledged. First, as with other PCR-based assays, the system is limited to predefined targets and cannot detect unknown or newly emerging GM events without prior sequence information. Second, although the current design successfully integrates 27 targets within a single reaction, further expansion of multiplexing capacity may increase primer–primer interactions and require additional optimization to maintain balanced amplification efficiency.

Nevertheless, these limitations do not affect its applicability in routine regulatory screening, where target information is generally predefined. Within this context, the proposed platform provides a practical, high-throughput, and reliable tool for simultaneous detection of multiple GM-related elements. Its single-reaction design and high-resolution capillary electrophoresis readout offer a useful balance between analytical efficiency, sensitivity, and operational simplicity, supporting its application in food safety monitoring, import/export inspection, and routine GMO surveillance.

## 5. Conclusions

In this study, we developed a multiplex fluorescent PCR–capillary electrophoresis platform for the simultaneous detection of multiple genetically modified maize-related targets, including endogenous genes, regulatory elements, and event-specific markers. The assay demonstrated high analytical performance, enabling accurate identification of multiple GM maize events, reliable discrimination of mixed samples, and a detection sensitivity of 0.1%. By enabling simultaneous detection of multiple genetic elements within a single analytical workflow, the method provides a practical tool for supporting routine GMO surveillance, regulatory inspection, and food safety monitoring in the context of rapidly increasing diversity of genetically modified organisms.

## Figures and Tables

**Table 1:** Primer sequences of Maize event Bt11.

Targets	Primer	Primer Sequence (5′–3′)
*Zein*	*Zein*-F	HEX-AGTGCGACCCATATTCCAGG
	*Zein*-R	TCACTGGCATCGTCTGAAGC
*pCaMV35S*	*CaMV35S*-F1	FAM-AGGAAAAGGAAGGTGGCTCC
	*CaMV35S*-R	AGAATAGTGGGATTGTGCGTCA
*NOS*	*NOS*-F1	FAM-GGTTTTTATGATTAGAGTCCCGC
	*NOS*-R1	AGTAACATAGATGACACCGCGC
*Cry1Ab*	*Cry1Ab*-F	FAM-CGTACTTGGTGGAGAACGCATTG
	*Cry1Ab*-R	GTTGAATACGCATTTCCTCGC
*Pat*	*Pat*-F	FAM-AGCAGCTGATATGGCCGC
	*Pat*-R	CAGCGTAAGCAATACCAGCC
Bt11	Bt11-F	AACCGTATTACCGCCTTTGAGT
	Bt11-R	HEX-ACAATAGCCCTCTGGTCTTCTGA

**Table 2:** Final concentration of primers.

Primer	Final Concentration/(μmol/L)	Primer	Final Concentration/(μmol/L)
*Zein*-F	0.033	*Cry1Ab*-F	0.22
Zein-R	0.033	*Cry1Ab*-R	0.22
*CaMV35S*-F1	0.096	*Pat*-F	0.24
*CaMV35S*-R	0.096	*Pat*-R	0.24
*NOS*-F1	0.06	Bt11-F	0.033
*NOS*-R1	0.06	Bt11-R	0.033

## Data Availability

The original contributions presented in this study are included in the article. Further inquiries can be directed to the corresponding author.

## Supplementary Materials

The following supporting information can be downloaded at https://www.mdpi.com/article/10.3390/cimb48070677/s1.
