# Supplementary material for: Multiplex PCR Fluorescence Method for Detection of Genetically Modified Maize Strains
Source: Curr Issues Mol Biol. 2026 Jun 30;48(7):677. doi: 10.3390/cimb48070677 (PMC13406978; doi:10.3390/cimb48070677)
Supplement: Supplementary file 1 [file cimb-48-00677-s001.zip › cimb-4371676-supplementary.pdf]

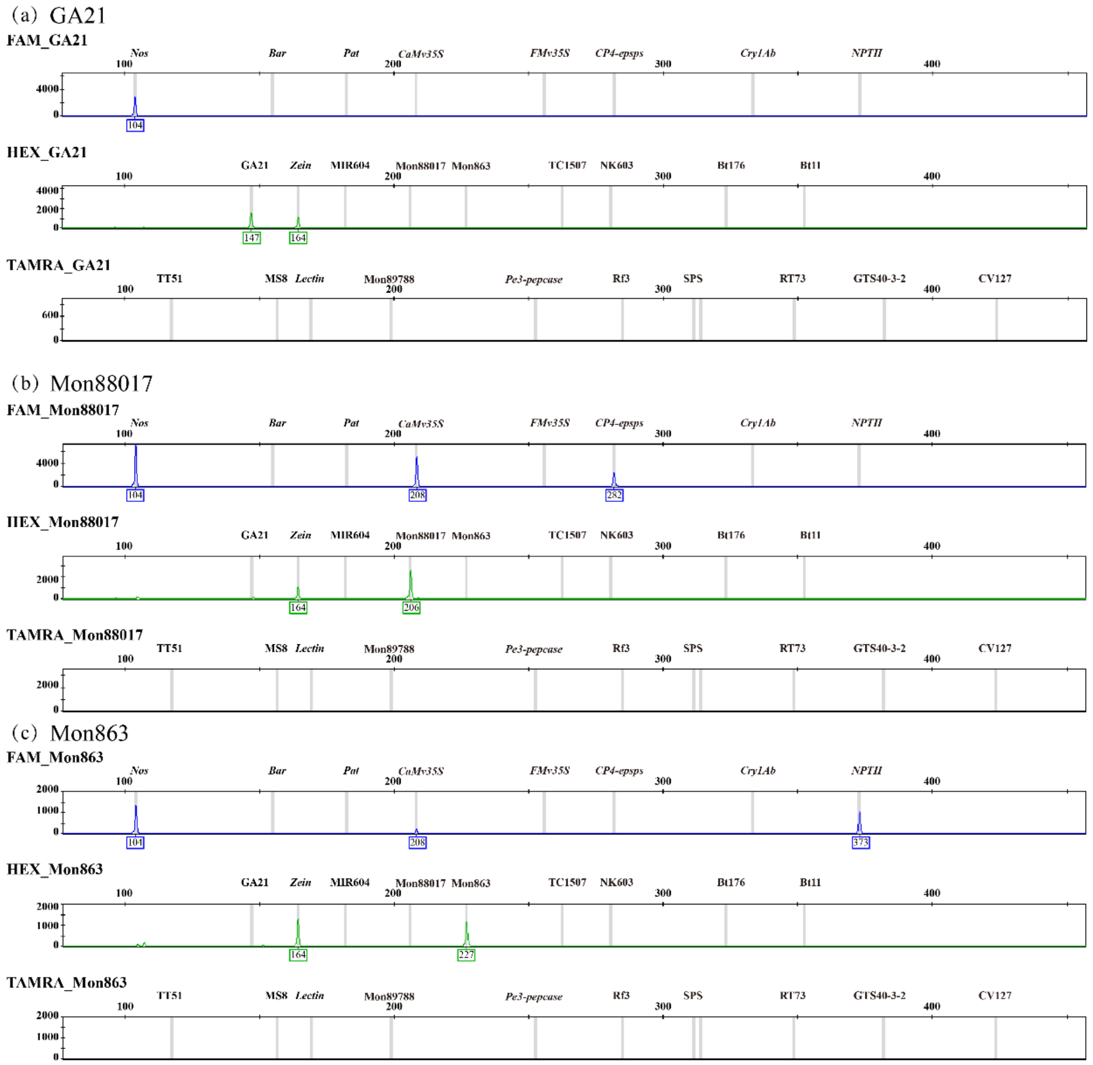

Supplementary Figure S1. Polymorphic fingerprints of three transgenic maize events: (a) GA21, (b) MON88017, and (c) MON863

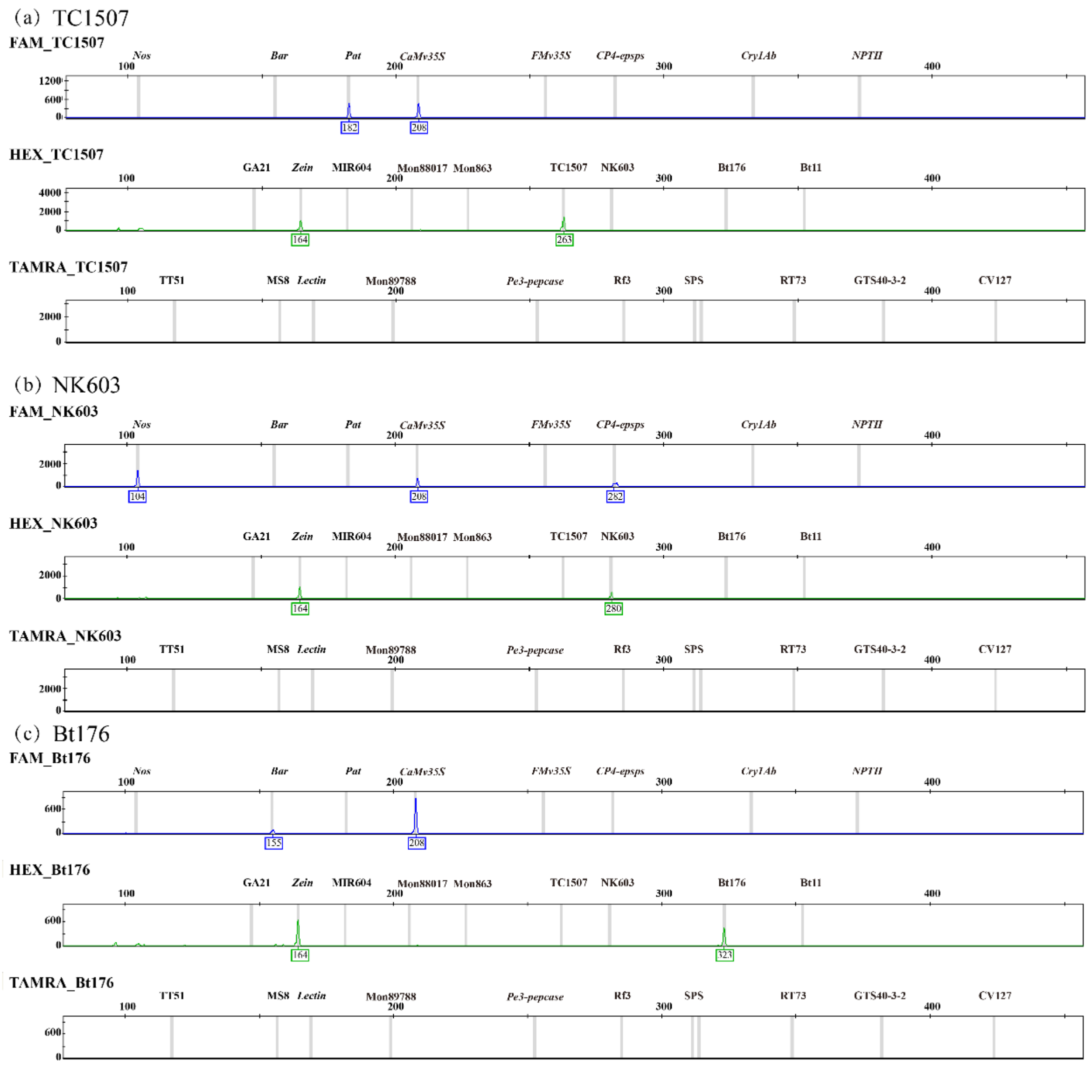

Supplementary Figure S2. Polymorphic fingerprints of three transgenic maize events: (a) TC1507, (b) NK603, and (c) Bt176

Table S1 Primer sequences of Maize event Bt11.

Gene	Primer	Primer sequence (5′-3′)
Bar	*Bar* -F<br>*Bar* -R	TCTACACCCACCTGCTGAAGTC<br>FAM-ACCAGTTCCCGTGCTTGA
FMv35S	*FMv35S*-F<br>*FMv35S*-R	FAM-GATTCTCAGTCCAAAGCCTCAAC<br>CGCCTAACAATTCTGCACCA
CP4-EPSPS	*CP4-EPSPS*-F<br>*CP4-EPSPS*-R	FAM-AGGTCTACGATTTCGACAGCA<br>GCATCTTTTCCGTATGATCGC
NPTII	*NPTII*-F<br>*NPTII*-R	FAM-GTCCTGCCGAGAAAGTATCCA<br>CGCCAAGCTCTTCAGCAATAT
GA21	GA21-nosF<br>GA21-nosR1	HEX-CGTTTTTATGATTAGAGTCCCGC<br>ATGGGGATCCACTAGTTCTAGAGC
MIR604	MIR604-F<br>MIR604-R1	HEX-GCGAACCAGTGAATGGAGATG<br>ATTGTCGTTTCCCGCCTTC
Mon88017	Mon88017/863-F<br>Mon88017-R	HEX-TAATCGGCTAATCGCCAACA<br>ACCATCCAAACCCGACTCTG
Mon863	Mon88017/863-F<br>Mon863-R1	HEX-TAATCGGCTAATCGCCAACA<br>GGACTTAGCCAGTTCATTGCG
TC1507	TC1507-F<br>TC1507-R	TCACGCATAGCACCTCATTCTT<br>HEX-ATGGTATTCTCTACCTACCCACCTG
NK603	NK603-F3<br>NK603-R	GTCTTCGAGTAAGCTTGTTAACGC<br>HEX-GCCGCATTACGAGCCATT
Bt176	Bt176-F<br>Bt176-R1	HEX-AACTGCCCGTCACCGAGAT<br>GATAGGGGACTTGACCGGAAG
TT51	TT51-F<br>TT51-R	TAMRA-GCGTCCAGAAGGAAAAGGAAT<br>AGACTGGTGATTTCAGCGGG
Ms8	Ms8-F<br>Ms8-R	GTGCCTTTTCTTATCGACCATG<br>TAMRA-GAAACTTGTGGGATGCTGTGG
Lectin	*Lectin*-F1<br>*Lectin*-R1	TAMRA-GCACCCAGAATGTGGTTGTATC<br>ACCCGAGGAGGTCACAATAGC
Mon89788	Mon89788-F<br>Mon89788-R	GTCGTGAAGTTTCTCATCTAAGCC<br>TAMRA-GCTTGAGGAGAGTGAAGGTGGT
Pe3-pepcase	*Pe3*-F<br>*Pe3*-R	TAMRA-CGCTTTGTTACAGGTCGCTATG<br>AAGAGAGGACGTTTCCCACTCA
Rf3	Rf3-F<br>Rf3-R	TAMRA-CAATAACTTTGTTGGGCTTATGG<br>CTCGTCTCGGACCTCCGAAAACC
Sps	*Sps*-F1<br>*Sps*-R1	TAMRA-ATTCAGTTTGTAACCACCGGATG<br>AGAGGAGTCCGTGGCTTGGT
Rt73	Rt73-F<br>Rt73-R	GCTTGTTTATTGCTTTCGCCTA<br>TAMRA-ACTTCTAGCCGTCGATTTCCA
GTS-40-3-2	GTS-40-3-2-F<br>GTS-40-3-2-R	TAMRA-ATGGCACAAGGGATACAAACC<br>ATGGCCTTGCCCGTATTG
CV127	CV127-F<br>CV127-R	GTCTAGCGATGAATTGGGTAGGT<br>TAMRA-GGCTCAACGGAAACTTCTGTT

Table S2 Final concentration of other primers.

Primer	Final concentration/ (μmol/L)	Primer	Final concentration/ (μmol/L)
FMv35S-F	0.064	TT51-F	0.20
FMv35S-R	0.064	TT51-R	0.20
CP4-EPSPS-F	0.18	Ms8-F	0.30
CP4-EPSPS-R	0.18	Ms8-R	0.30
NPTII-F	0.18	*Lectin*-F1	0.064
NPTII-R	0.18	*Lectin*-R1	0.064
GA21-nosF	0.18	Mon89788-F	0.06
GA21-nosR1	0.18	Mon89788-R	0.06
MIR604-F	0.20	*Pe3*-F	0.24
MIR604-R1	0.20	*Pe3*-R	0.24
Mon88017/863-F	0.15	Rf3-F	0.24
Mon88017-R	0.05	Rf3-R	0.24
Mon863-R1	0.30	*Sps*-F1	0.07
TC1507-F	0.24	*Sps*-R1	0.07
TC1507-R	0.24	Rt73-F	0.20
NK603-F3	0.25	Rt73-R	0.20
NK603-R	0.25	GTS-40-3-2-F	0.30
Bt176-F	0.26	GTS-40-3-2-R	0.30
Bt176-R1	0.26	CV127-F	0.20
		CV127-R	0.20
